# Fractionated External-beam Radiation Therapy For Incompletely Resected Intracranial Extra-axial Cavernous Haemangioma: A Case Report

**DOI:** 10.7759/cureus.2285

**Published:** 2018-03-07

**Authors:** Kiat Huat Ooi, Shiong Wen Low

**Affiliations:** 1 National University Cancer Institute, National University Hospital Singapore; 2 Neurosurgery, Ng Teng Fong General Hospital

**Keywords:** intracranial, cavernoma, radiotherapy, external-beam, cavernous hemangioma, cavernous haemangioma, extra-axial, radiation therapy

## Abstract

We report a case of a cavernous haemangioma arising from the left trigeminal nerve causing mass effect, midline shift and hydrocephalus. Surgical resection was attempted after immediate insertion of bilateral extra-ventricular drains, but complete resection was not achieved, with residual disease at the superior orbital fissure and cavernous sinus.

Stereotactic radiosurgery was deemed unsuitable in view of proximity to the left optic nerve and optic chiasm, and the patient was hence treated with fractionated external-beam radiation therapy (RT) to a total dose of 40 Gy in 20 daily fractions over four weeks.

The patient tolerated RT well, with no significant toxicity. MRI done eighteen months after completion of RT showed the continued decrease in the size of the lesion with reduced mass effect on the optic chiasm.

## Introduction

Intracranial cavernous haemangiomas are estimated to comprise 0.1–4% of all vascular malformations of the brain [1]. They are mostly intra-axial in origin, and extra-axial cavernous haemangiomas are rare [2]. Unlike the former, extra-axial cavernous haemangiomas rarely present with haemorrhage and more commonly present with cranial nerve deficits and mass effect [3]. The established treatment for extra-axial cavernous haemangiomas is neurosurgical excision or stereotactic radiosurgery [4], but in the event that both these modalities are not possible, fractionated external-beam radiation therapy may be an option. We report such a patient treated with fractionated external-beam radiation therapy to a total dose of 40 Gy in 20 daily fractions over four weeks, with good response on post-treatment imaging.

## Case presentation

A 28-year-old female with no significant medical history was brought to the emergency department for drowsiness and unresponsiveness to calling. This was associated with three episodes of non-bilious and non-bloody vomiting. On further questioning of her housemates, she had been complaining of recurrent headaches and drowsiness for about two months prior. On examination, she was found to have a reduced level of consciousness, with a Glasgow Coma Scale (GCS) score of eight (E2V1M5). Her bilateral pupils were equal at 4 mm and reactive to light. The rest of the examination at that time was limited in view of her decreased GCS, but unremarkable.

**Figure 1:**
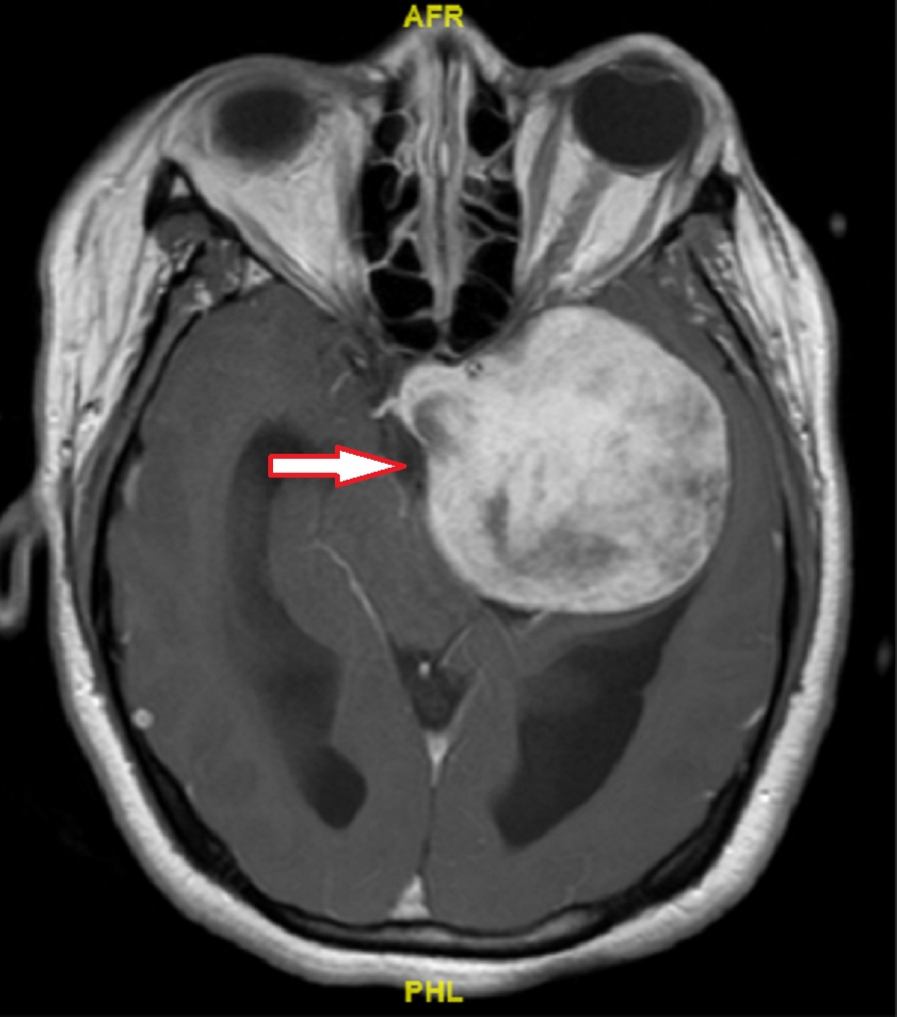
Initial T1 Contrast-enhanced Magnetic Resonance Imaging Axial view of tumour (arrow)

**Figure 2:**
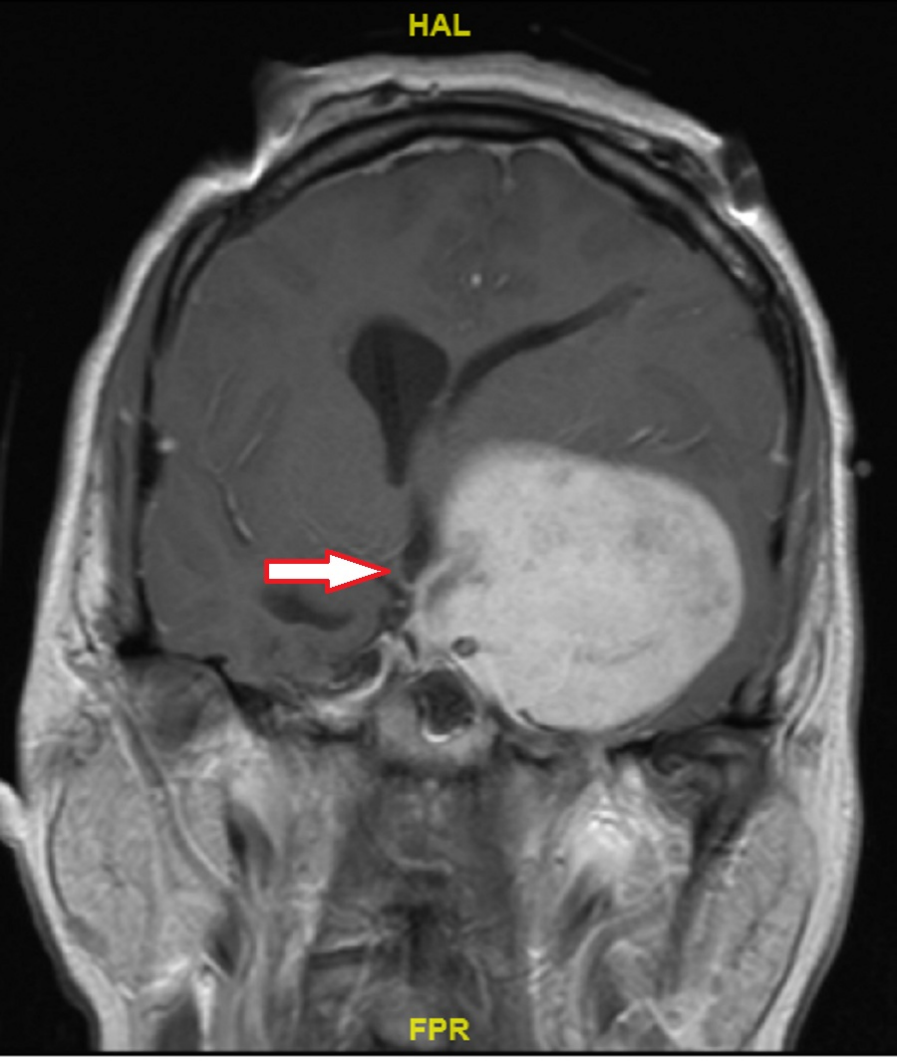
Initial T1 Contrast-enhanced Magnetic Resonance Imaging Coronal view of tumour (arrow)

**Figure 3:**
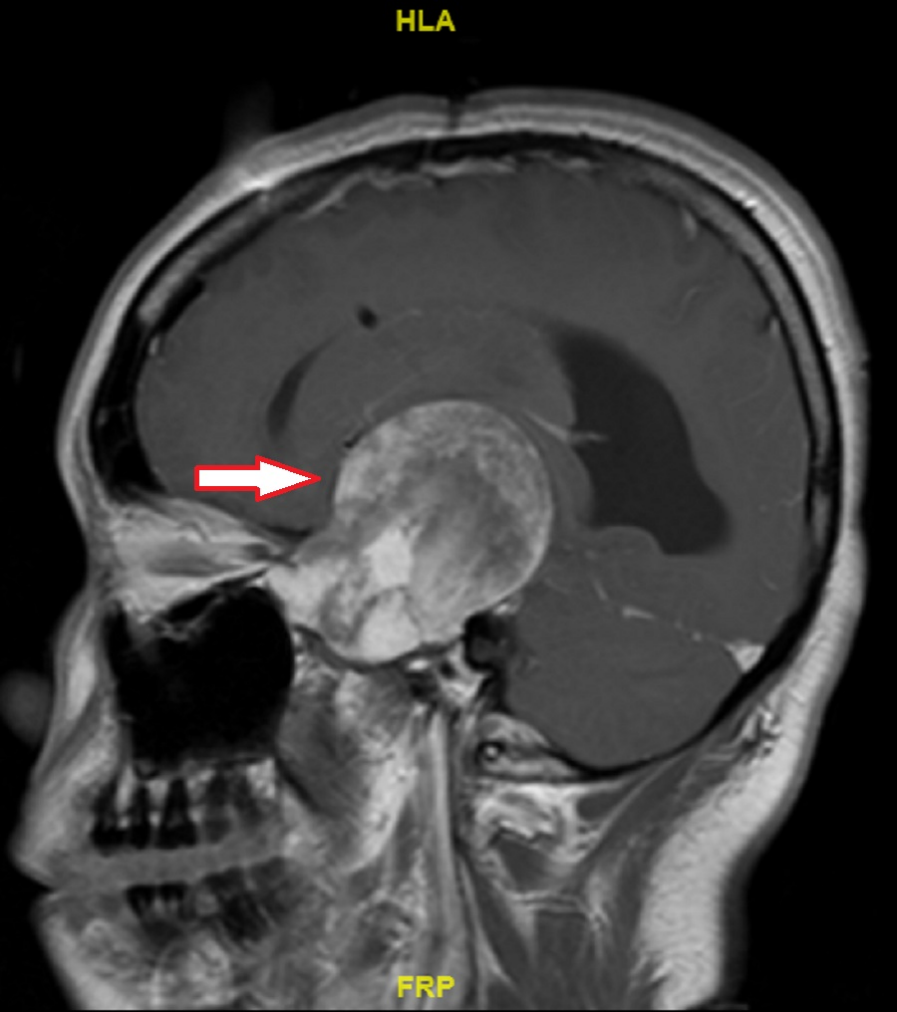
Initial T1 Contrast-enhanced Magnetic Resonance Imaging Sagittal view of tumour (arrow)

Magnetic resonance imaging (MRI) scan of the brain (Figure 1, Figure 2 and Figure 3) characterized the lesion as a left middle cranial fossa extra-axial mass measuring 6.4 x 5.8 x 5.7 cm, appearing to arise from the floor of the left sphenoid wing. This lesion was noted to be causing a 1.4 cm midline shift to the right, obstructive hydrocephalus, and possible impingement upon the left optic nerve. At the time, it was reported to be suspicious for a meningioma.

**Figure 4:**
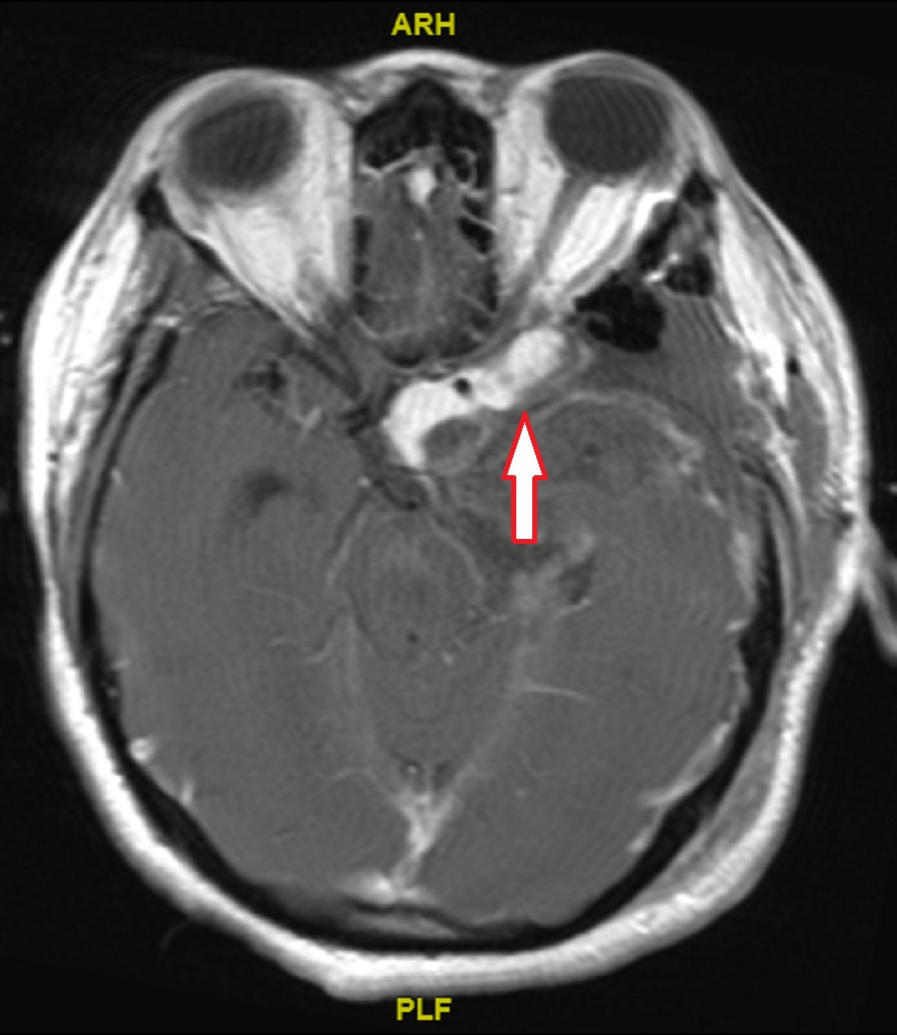
Post-surgery T1 Contrast-enhanced Magnetic Resonance Imaging Axial view of tumour, demonstrating residual tumour after surgery (arrow)

The patient underwent immediate insertion of bilateral extra-ventricular drains, following which her GCS improved to 15. She was also noted to have palsies of her left third and fourth cranial nerves (CN III and IV). She subsequently underwent a craniotomy and resection of the left middle cranial fossa mass. Intraoperatively, the lesion was noted to be very vascular, and arising from the left trigeminal nerve. Of note, the lesion was found to be infiltrating into the cavernous sinus and left superior orbital fissure, and it was at these two sites that residual disease was left behind. A repeat MRI scan subsequently confirmed residual disease measuring 1.4 cm x 3.8 cm at this area (Figure 4).

Histopathological examination of the resected specimen revealed variably dilated, thin-walled blood vessels with no brain or glial tissue incorporated in the tissue between the lesional blood vessels. This was diagnosed as a cavernous haemangioma.

Gamma-knife was considered for treatment of the residual disease but was deemed unsuitable due to the proximity of the optic apparatus. Stereotactic radiation therapy (SRT) was discussed with the patient but declined in view of treatment costs. The patient was hence treated with fractionated external-beam radiation therapy (RT) to a total dose of 40 Gy in 20 daily fractions over four weeks.

**Figure 5:**
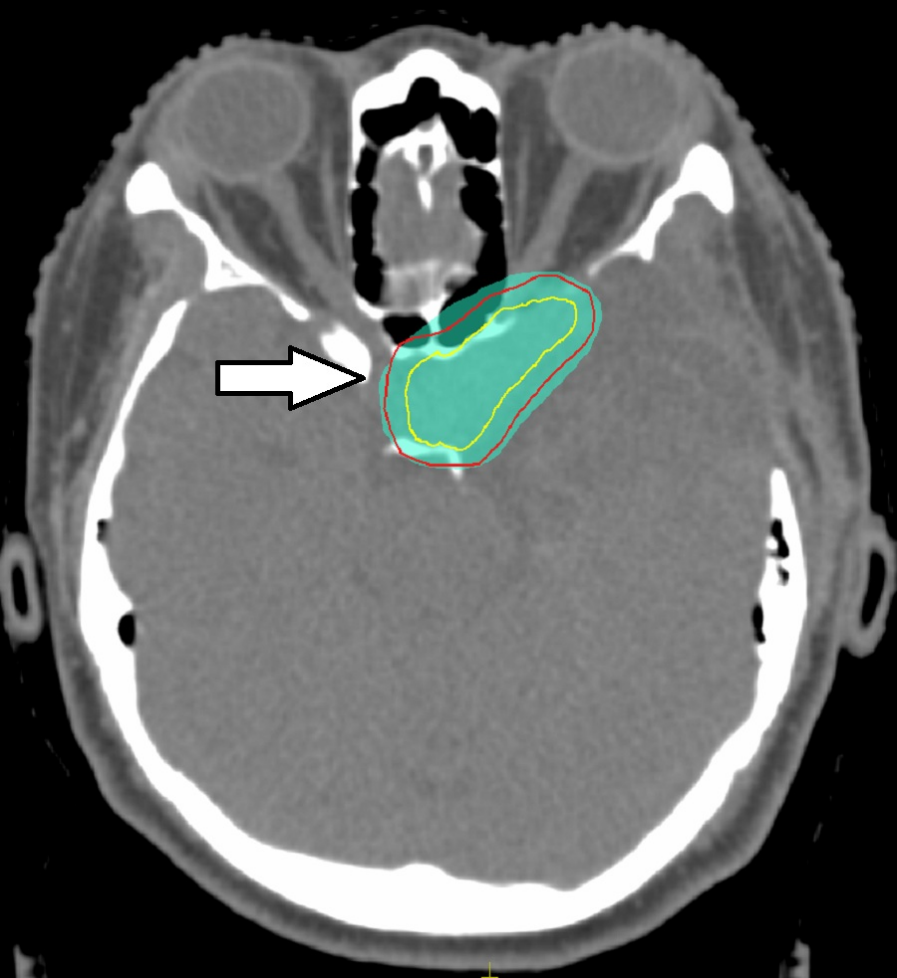
Representative Image of Radiation Therapy Plan Gross Tumour Volume (solid yellow line) and Planning Target Volume (solid red line) encompassed by the 95% isodose (green colourwash) (arrow).

The treatment was computed tomography (CT)-planned, with gross tumour volume (GTV) being the residual disease seen on imaging. The GTV was directly expanded (no clinical target volume expansion) by 5 mm to form the planning target volume (PTV), which was encompassed by at least 95% of the prescribed dose. The patient was treated with an intensity-modulated radiation therapy (IMRT) technique, utilizing six mega-voltage (MV) photons. A representative image of the RT plan is shown in Figure 5. The maximum dose to the pituitary gland was less than 43 Gy.

**Figure 6:**
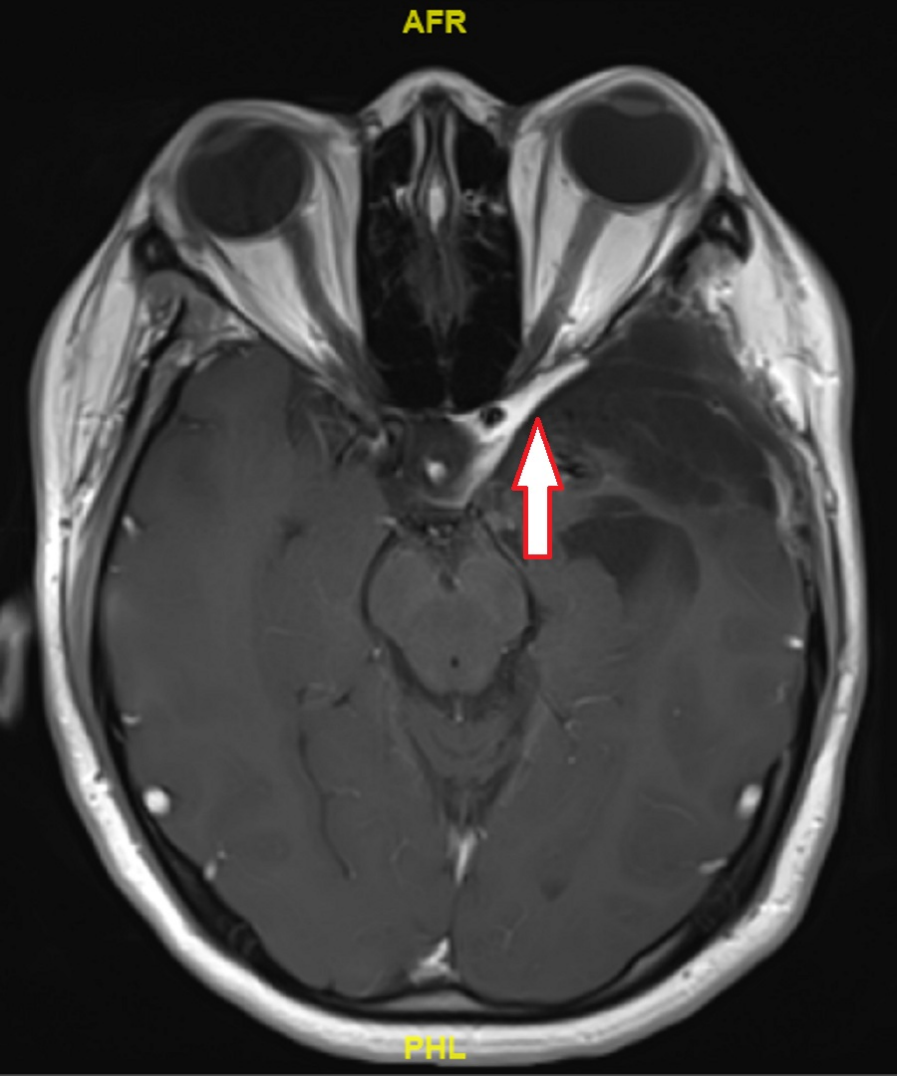
Eighteen months post-Radiation Therapy T1 Contrast-enhanced Magnetic Resonance Imaging Axial view of tumour, demonstrating reduction in size when compared with the immediate post-surgery scan (arrow)

The RT was well tolerated, and the patient did not experience any significant toxicity. MRI scan done five months after completion of RT (Figure 6) showed an interval decrease in the size of the residual disease, from 1.4 cm x 3.8 cm to 0.8 cm x 2.9 cm, and decreased mass effect on the optic chiasm. Subsequent scans showed the continued reduction in the size of the lesion, with the most recent MRI done eighteen months after completion of RT showing a residual rind of enhancing soft tissue persisting in the left para-sellar region and along the anterior temporal fossa measuring 3.1 cm x 0.4 cm. Clinically, she remains asymptomatic apart from left eye ptosis and ophthalmoplegia (which have been present post-surgical resection), with excellent functional status.

## Discussion

While there have been reports in the literature on the use of fractionated radiation therapy for intracranial cavernous haemangiomas, these have been limited to small case series [5-7] utilizing a range of doses, from 30 Gy to 50.4 Gy. In our patient, the proximity of the lesion to the pituitary gland provided additional impetus to seek the lowest effective dose, in a bid to reduce the likelihood of hypituitarism as a late effect of radiation.

Our patient was treated with fractionated external-beam radiation therapy to a total dose of 40 Gy in 20 daily fractions over four weeks, which resulted in an objective and sustained reduction in the size of the residual lesion. She did not experience any significant acute radiation toxicity during the course of treatment, and has not demonstrated any late radiation toxicity thus far. Radiation-induced optic neuropathy is unlikely to develop, as the dose of radiation therapy delivered is well within the established tolerance for the optic apparatus, and she will continue to be monitored for hypopituitarism.

## Conclusions

Intracranial extra-axial cavernous haemangiomas are rare. The treatment of choice for these lesions is either neurosurgical excision or stereotactic radiosurgery. Our patient initially underwent a subtotal resection, with gross residual disease. This was successfully treated with external-beam radiation therapy. Hence, we surmise that in situations where complete neurosurgical excision or stereotactic radiosurgery are not feasible, fractionated external-beam radiation therapy can be an effective treatment for extra-axial cavernous haemangioma. The dose that we utilized (40 Gy in 20 fractions over four weeks) appears to be effective, but the optimal dose is yet to be established and should be further investigated.
